# Micro RNA Sensing with Green Emitting Silver Nanoclusters

**DOI:** 10.3390/molecules25133026

**Published:** 2020-07-02

**Authors:** Liam E. Yourston, Alexey V. Krasnoslobodtsev

**Affiliations:** Department of Physics, University of Nebraska Omaha, 6001 Dodge Street, Omaha, NE 68182, USA; lyourston@unomaha.edu

**Keywords:** silver nanoclusters, miR detection, fluorescence, cytosine rich sequences, miR-21

## Abstract

Micro RNA (miR) are regulatory non-coding RNA molecules, which contain a small number of nucleotides ~18–28 nt. There are many various miR sequences found in plants and animals that perform important functions in developmental, metabolic, and disease processes. miRs can bind to complementary sequences within mRNA molecules thus silencing mRNA. Other functions include cardiovascular and neural development, stem cell differentiation, apoptosis, and tumors. In tumors, some miRs can function as oncogenes, others as tumor suppressors. Levels of certain miR molecules reflect cellular events, both normal and pathological. Therefore, miR molecules can be used as biomarkers for disease diagnosis and prognosis. One of these promising molecules is miR-21, which can serve as a biomarker with high potential for early diagnosis of various types of cancer. Here, we present a novel design of miR detection and demonstrate its efficacy on miR-21. The design employs emissive properties of DNA-silver nanoclusters (DNA/AgNC). The detection probe is designed as a hairpin DNA structure with one side of the stem complimentary to miR molecule. The binding of target miR-21 opens the hairpin structure, dramatically modulating emissive properties of AgNC hosted by the C_12_ loop of the hairpin. “Red” fluorescence of the DNA/AgNC probe is diminished in the presence of the target miR. At the same time, “green” fluorescence is activated and its intensity increases several-fold. The increase in intensity of “green” fluorescence is strong enough to detect the presence of miR-21. The intensity change follows the concentration dependence of the target miR present in a sample, which provides the basis of developing a new, simple probe for miR detection. The detection strategy is specific, as demonstrated using the response of the DNA/AgNC probe towards the scrambled miR-21 sequence and miR-25 molecule. Additionally, the design reported here is very sensitive with an estimated detection limit at ~1 picomole of miR-21.

## 1. Introduction

Silver nanoclusters (AgNC) are a novel class of nanomaterials that are only a few atoms in size. Cytosine-rich nucleic acid sequences template and stabilize silver nanoclusters limiting their size and modifying their electronic properties. The continuous density of states, typical for metal structures, breaks up into discrete energy levels in the nanoclusters. The nanoclusters resemble molecular-like behavior with strong fluorescence observed in the wide range of the spectrum, from UV to visible to near IR [1,2]. AgNCs are more stable in photobleaching than widely used organic dyes, quantum dots, or fluorescent proteins [3,4,5]. These properties make AgNCs suitable for a plethora of practical applications including luminescent labelling, biological imaging, sensing, and catalysis [6,7,8]. The high sensitivity of emissive properties of the AgNCs to subtle changes in their environment have triggered an exploration of practical use of the AgNCs in sensing applications. Since the precise knowledge of metal nanocluster photophysics is still lacking, further understanding of the energy transfer mechanism when utilizing metal nanoclusters is expected to result in a broader interest of utilizing nanoclusters in biosensing applications [9].

The use of DNA-templated silver nanoclusters has been proposed for the detection of a variety of species based on fluorescence quenching or fluorescence enhancement. For example, sulfide S^2-^ ions have been effectively detected [10]. DNA-templated silver nanoclusters have been shown to be responsive to the presence of various metal ions. For example, the sensing of Cu^2+^ [11,12,13] was demonstrated with the use of silver nanoclusters hosted in the polymethyl methacrylate (PMMA) network [14] or glutathione (GSH) ligand [15], Hg^2+^ was detected with DNA/AgNCs [16,17,18,19,20]. The sensitivity of AgNCs to ions has also been exploited in the construction of logic gates [21,22]. A variety of already-developed approaches indicate how versatile the use of AgNCs can be for the detection of a single analyte.

Not only inorganic analytes can be sensed with silver nanoclusters, but also the detection of biomolecules has been explored by several groups. Among examples of biomolecular detection are gene detection [23], protein detection [24,25,26], detection of specific nucleic acid sequences [27,28,29], and cellular labeling [30,31]. Recently, advanced detection procedures have also been incorporated with the use of AgNCs such as the multiplexed sensing capability of several genes [32] or surface plasmon enhanced energy transfer (SPEET) between AgNCs and AuNP for protein [33] or DNA [34] detection.

The detection of miR molecules represents an enormous interest, owing to their important role in many processes. miR molecules are regulatory non-coding small sequences comprising a limited number of nucleotides ranging between ~18–28 nt. miR molecules play important roles in developmental, metabolic, and disease processes. Of particular interest is the role miR play in tumors. Some miR molecules can function as oncogenes, others as tumor suppressors [35]. Dysregulation and the extent at which miR molecules are present in cells can serve as a disease fingerprint. Since levels of certain miRs reflect pathological cellular events, these miR molecules can be used as biomarkers for disease diagnosis and prognosis. One of such promising molecules is miR-21 which is a representative oncogenic miR so called “onco-miR”. miR-21 is a 22 nucleotides-long molecule that drives cancer development by targeting numerous tumor suppressor genes associated with proliferation, apoptosis and invasion including PTEN [36], PDCD4 [37,38], and TPM1 [39]. miR-21 is overexpressed in various types of tumors. The following are examples of confirmed roles of the miR-21 in tumors: breast [40], lung [41], ovarian [42], cervical [43,44], colon [38], brain [45,46], liver [47,48], prostate [49], and pancreatic cancer [50,51]. Dysregulation of miR-21 makes it a very good candidate as a biomarker for developing tools for the early diagnosis and prognosis of cancers. More importantly, as recent evidence suggests, miR-21 is not only present in tissues but also in body fluids, such as serum [52,53], saliva [54,55], plasma [46,56,57], gastric juice [58,59], sputum [60,61], and cerebrospinal fluid [62,63,64]. Therefore, by examining body fluids and the levels of miR-21, it may be possible to differentiate cancer patients from healthy individuals and detect cancer at early stages dependent on the diagnostic potential of miR-21 as a biomarker. Thus, new methods for the rapid and sensitive detection of miRs are of paramount importance in the field of cancer diagnostics.

Recently, several designs utilizing DNA-based AgNCs have been exploited for the detection of various miR sequences [27,28,29,65]. The detection strategy for miR molecules is primarily based on a clever construction of the AgNC probe which involves two separate domains: the recognition domain and the signaling domain. The signaling domain templates and stabilizes AgNCs while the recognition domain specifically binds to biomolecules needed to be detected: specific gene sequences, protein aptamers, and nucleic acid sequences such as miR. The two domains work concertedly due to the high sensitivity of AgNCs to changes in the environment. It is important for the sensitivity of detection that the signaling domain responds to slight variations in the environment upon analyte binding to the recognition domain. Signal changes can be positive when fluorescence is enhanced due to analyte binding or negative when fluorescence is quenched. The ability of the AgNCs to detect short fragments of nucleic acid molecules stems from convenient natural integration of AgNCs into nucleic acid-based nano-assemblies [2]. We dedicated this current study to demonstrating the detection strategy for miR sequences in general and miR-21 in particular. Here, we present a novel type of miR detection in cases where signaling modality is due to the activation of dark fluorescence states, which remain silent in the absence of a target analyte but become activated when the analyte is present. We evaluated the response of a hairpin-based biosensor to the presence of miR-21 in a sample. The sensor design contained a C_12_ loop that effectively templated AgNCs with a bright “red” emission and high stability. In the presence of miR-21 molecules, a new “green” emission appears and its intensity increases in an miR-21 concentration-dependent manner. The novelty of the design presented here exploits activation of the “green” emissive states only due to specific binding of the miR sequence, providing the selective fluorescence contrast enhancement mechanism of miR detection. While the major goal of this work was to demonstrate the feasibility of detection and evaluate the sensitivity of the loop-templated fluorescent AgNC towards a particular miR sequence, miR-21, the results of this study and the design presented can easily be generalized for other miR sequences.

## 2. Results and Discussion

### 2.1. Design of Sensing AgNC DNA Template

There is a variety of DNA sequences that template and stabilize silver nanoclusters (reviewed in [66]). Recently, it has been proposed that the identification of specific genome sequences can be made based on the properties of AgNC that these sequences template [67]. The cytosine-rich sequences are the most common nucleic acid sequences used in the synthesis of AgNC due to the very high affinity of cytosines to silver cations [68,69]. Our primary goal is to be able to change optical properties of silver nanoclusters upon binding of a short RNA sequence of interest corresponding to a specific type of miR.

**Figure 1:**
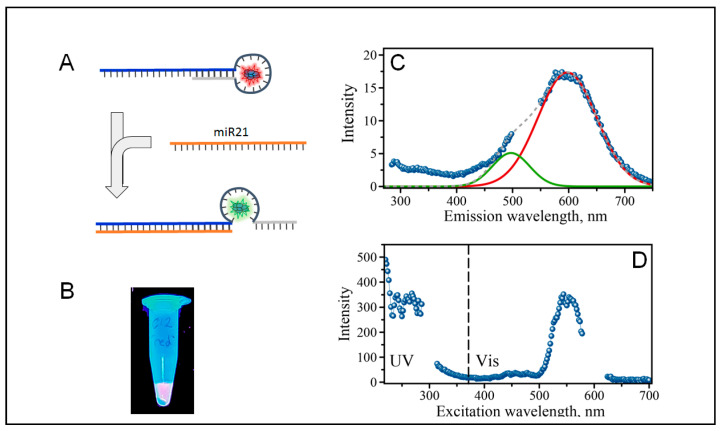
(**A**) Schematic representation of the template design and detection strategy, (**B**) a photograph of fluorescent glowing of silver nanoclusters (AgNCs) templated on C_12_-loop-miR21-probe under the UV excitation on trans-illuminator. (**C**) Fluorescence spectrum of AgNCs/C_12_-loop-miR21-probe recorded with 260 nm excitation wavelength, (**D**) Excitation spectrum of the AgNCs/C_12_-loop-miR21-probe for the emission at λ_R_ = 600 nm. Gaps in the spectra are due to removal of the second order scattering. Green and red solid lines are plotted as “guide for the eye” in the positions of major “green” and “red” emission peaks.

A DNA/AgNC probe was designed as a hairpin template to contain two domains. One domain is a cytosine rich loop to host AgNCs. The second domain is a stem of the hairpin which is partly complementary to target miR molecule. Figure 1A schematically shows our design. The designed sequence has a double-stranded stem and carries the nanocluster nucleating C_12_ loop. One side of the stem sequence is a full-length complementary sequence to miR-21. In the presence of miR-21, the longer side of the design binds miR-21 due to perfect complementarity of the two sequences. The other side of the stem is a seven-base-long segment and is complementary to the bases of the stem right next to the C_12_ loop (TAGCTTA). Thermodynamic calculations suggest that the stability of the full-length, 22 bp compliment (∆G = −35.09 kcal/mole) is larger than stability of the 7 bp stem duplex (∆G = −2.92 kcal/mole). Due to such differences in thermodynamic stability of these two duplexes, the operation of the designed miR probe is expected to optimally proceed at room temperature. The design of the stem, length and the sequence can be made more or less stable depending on the probed miR sequence—rendering the proposed design flexible and suitable for a variety of different sequences.

### 2.2. The Formation of Silver Nanoclusters within the C_12_ Loop of the DNA-miR21-Probe

The formation of fluorescent silver nanoclusters was templated by the C_12_ loop of the C_12_-loop-miR21-probe, which assumes a hairpin structure (Figure 1A). The AgNC formation is manifested by the observable changes in solution. Within just a few hours after the addition of AgNO_3_ and sodium borohydride as reducing agent to the C_12_-loop-miR21-probe, the solution loses transparency and acquires a clear reddish tint. These changes can be observed even with the naked eye but become more apparent when the sample is placed under a UV trans-illuminator (Figure 1B). The samples glow red under these conditions. Figure 1C shows an emission spectrum of the sample with constant single excitation at 260 nm mimicking excitation into DNA bases [2]. As expected, the spectrum is dominated by the red emission with an apparent maximum at λ_R_ = 600 nm. Although the major peak is situated at λ_MAX_ = 600 nm, one can notice a shoulder at lower wavelengths that might suggest the presence of “green” fluorescence as well. We have previously observed both types of emitters “red” and “green” coexisting with C_12_ templating sequences [2], although it appears that the C_12_-loop of the hairpin primarily gives rise to “red” emitters. Figure 1D shows an excitation spectrum of the red emission, λ_R_ = 600 nm. The excitation spectrum reveals that the red fluorescence can be excited in the visible with broad excitation peak at λ_EXC_ = 510–590 nm range. It is also broadly excited throughout the UV region, covering an astonishing range of 220–315 nm.

### 2.3. Optical Properties of AgNCs/C_12_-loop-miR21-Probe under UV and Visible Excitation

We further characterized the properties of AgNC templated with DNA-miR21-probe by employing fluorescence excitation–emission matrix spectroscopy (EEMS). The optical response and the excitation/emission relationship of AgNCs has proven to be a little complicated, hence a better way to represent such responses is through measuring the entire excitation/emission matrix spanning a wide range of wavelengths presented as 2D contour maps [2,70,71].

**Figure 2:**
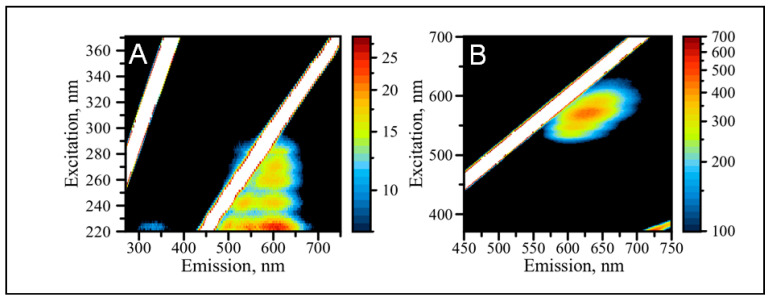
Detailed excitation-emission maps of AgNC/C_12_-loop-miR21-probe. (**A**) UV/UV-Vis excitation-emission map and (**B**) Vis/Vis excitation-emission map.

Figure 2A shows the EEM map for the AgNCs templated on C_12_-loop-miR21-probe with excitation in the range of 220–370 nm while recording the entire emission spectrum in the UV and visible spanning 270–750 nm wavelengths (UV/UV-Vis EEM). Similar to the spectrum with single wavelength of excitation shown in Figure 1C, the red emission dominates the map with the most intense fluorescence at 600 nm. “Red” fluorescence is excited broadly throughout the entire UVC (220–280 nm) and UVB (280–310 nm) regions. There is also detectable amount of “green” fluorescence in the emission range of wavelengths between 500–600 nm. The emission is not excited continuously throughout these regions for both “green” and “red” species but rather with a set of wavelengths: 225, 240, 260, and 274 nm, determined as maxima of the Gaussian fits. Regardless of color, the emission of AgNCs in the visible is universally excited via the DNA bases which agrees well with previously published data [72] and our own observations [2]. Previous studies found that visible emission of DNA templated AgNCs can be excited by both direct excitation into visible band and by UV light into absorption peak of DNA nucleobases [2,69,73]. The tight association of silver nanoclusters with DNA templating bases allows for optical interactions between the DNA template and AgNCs [72]. Therefore, in addition to natural integration of the template into a detection probe design, the nucleobases’ targeted excitation of normal emission bands of AgNCs is another valuable feature of the DNA template.

Next, we measured excitation/emission map for the AgNCs templated on the C_12_-loop-miR21-probe with excitation in the visible range between 370 nm and 700 nm while also recording the visible emission spectrum spanning 450–750 nm wavelengths (Vis/Vis EEM), shown in Figure 2B. The Vis/Vis EEM probes optical properties of the nanoclusters in general, despite their association with the bases. This is in contrast to UV/UV-Vis EEM, which reveals properties of the AgNCs tightly associated with the nucleobases. It is clear that the “red” emission is dominant in the Vis/Vis EEM. The red emission appears as diagonally stretched peak extending from λ_EM_ = ~580 nm to λ_EM_ = ~680 nm. The diagonal pattern of the “red” emission is related to the progressive shift of the maximum wavelength to the red as the excitation wavelength increases. Such bathochromic shifting in the emission band is most likely associated with the Red-Edge Excitation Shift (REES) effect [74]. This phenomenon has been previously observed for polar fluorophores in “rigid” solutions [75,76,77]. We hypothesize that such an effect in AgNC indicates a “rigid” nature of clusters, preventing fast randomization of the local environment. The randomization process is not fast enough to allow for efficient relaxation of the excited state of the cluster to the lowest possible level. The emission is forced from the higher levels of the excited singlet state resulting in the shift of emission to the red with increasing excitation energy [74].

In general, we find that “green” and “red” emissions are not equivalent for the excitation for both UV and visible. Despite its detectable intensity when excited in UV, the “green” emission is silent when excitation is performed in the visible range of wavelengths. Additionally, the “red” peak appears at a different maximum wavelength when compared to UV excitation. Although with complex excitation pattern, UV excitation results in a single maximum of emission λ_RED-MAX_ = 600 nm (λ_EXC_/λ_EM_ = 225/600 nm, λ_EXC_/λ_EM_ = 240/600 nm, λ_EXC_/λ_EM_ = 260/600 nm, λ_EXC_/λ_EM_ = 274/600 nm). The fluorescence under visible excitation features several peaks. While the maximum of emission starts at 600 nm with a small peak λ_EXC_/λ_EM_ = 535/600 nm, it progressively shifts to red and outlines a complex de-excitation pattern for the red emission. The preferred emission is observed at λ_EXC_/λ_EM_ = 570/628 nm and highlights the differences between UV and visible excitation, while both show a complex pattern, UV does not have REES while visible excitation shows obvious REES. This is similar to what we observed before for the red emission of many C_12_ templated AgNCs, and indicates a complex nature of the energy landscape as well as an assortment of de-excitation routes of the AgNC emissive states.

### 2.4. Detection of miRNA-21 with “Green” AgNCs

Close proximity of the bases coordinating the AgNCs affect electronic properties and alter photophysics—both emission and excitation, as well as quantum yield and fluorescence lifetime [78]. While it is still not fully understood how exactly templating and non-templating sequences affect the photophysics of AgNCs, it is nonetheless possible to utilize these properties for some practical applications. The sensitivity of optical properties of AgNCs to their environment can be used to detect miR sequences. We employed the C_12_-loop-miR21-probe for detecting the presence of miR-21 molecule added to the sample.

**Figure 3:**
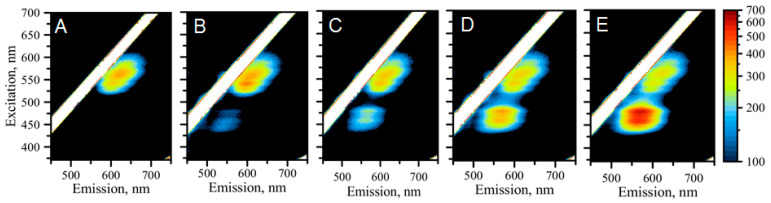
The collection of 2D spectra showing changes upon progressive addition of molar equivalent of miR-21 to C_12_-loop-miR21-probe with AgNCs. (**A**) 0 molar equivalent; (**B**) 0.25 molar equivalent; (**C**) 0.50 molar equivalent; (**D**) 0.75 molar equivalent; and (**E**) 1.00 molar equivalent.

To assess the feasibility to detect and quantitatively report on the presence of miR molecules, we measured the EEM spectra for a series of samples with varying concentrations of target miR-21 molecules. Figure 3 shows the response of the AgNCs/C_12_-loop-miR21-probe emission to addition of molar equivalents of miR-21 molecules to C_12_-loop-miR21-probe in the following order 0, 0.25, 0.5, 0.75, 1.00 with a 25% incremental increase in the molar ratio (M_miR21_/M_AgNC_). The results showed that the optical response of the AgNCs/DNA-probe is sensitive to the presence of the miR-21 molecule in a concentration-dependent manner. The original red emission does not change dramatically as indicated by the same pattern with the fine structure of subtle peaks. Perhaps the only thing that changes is the relative intensity of the peaks. While the entire cluster of “red” emission peaks shows a similar intensity regardless of how much of the miR-21 target molecule is added, it appears that satellite peaks on the green edge of the red cluster become intensified when miR-21 is added to the C_12_-loop-miR21-probe.

**Figure 4:**
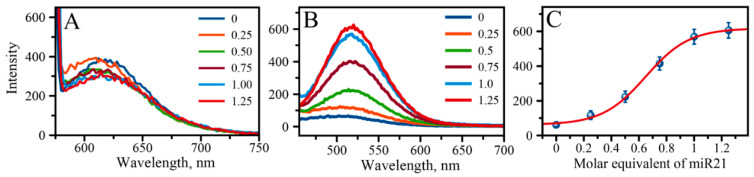
(**A**) Comparative graph of emission spectra for the “red” AgNCs excited at λ = 560 nm showing spectral changes upon incremental addition of molar equivalent of miR-21 to AgNC/C_12_-loop-miR21-probe; (**B**) Comparative graph of emission spectra for the “green” AgNCs excited at λ = 480 nm showing spectral changes upon incremental addition of molar equivalent of miR-21 to AgNC/C_12_-loop-miR21-probe; (**C**) Fluorescence intensity plot, λ_EXC_/λ_EM_ = 480/565 nm, as a function of miR21 molar equivalent concentration showing the fit (red solid line) using Equation (1), error bar represents variations based on three independent measurements.

The most dramatic change, however, is observed in the “green” region of emission spectra, at λ_EM_ = 565 nm. The intensity of the “green” emission increases with increased concentration of miR-21 suggesting that the high sensitivity of this type of AgNC is attributable to changes due to the presence of an analyte sequence. As it turns out, the “green” emission is also complex and represents itself as a cluster of “green” peaks with all the peaks in the cluster increasing similarly in intensity with the increase of the analyte concentration. Therefore, it is possible to utilize the increase in fluorescence intensity as a read-out strategy for the detection and quantitative analysis of miR-21. Figure 4A shows the comparative graph of fluorescence spectra all excited at λ_EXC_ = 570 nm, demonstrating that the “red” emission has very poor responsive changes to various concentrations of miR-21. The maximum of the emission spectrum slightly shifts and only marginally drops when the miR-21-equivalent concentration is changed from 0 to 0.25 and then to 0.5, and after that it stays almost the same at high molar equivalents of miR-21 added. Such behaviour invalidates the possibility of using “red” emission as a read-out signal for the quantitative analysis of miR-21. On the other hand, steep changes in intensity of “green” fluorescence may be, in fact, considered as a detection read-out method. Indeed, as shown in Figure 4B, the maximum intensity of the peak plotted versus the concentration of added miR-21 exhibits a sigmoidal behaviour: the larger the amount of added analyte sequence, the larger the intensity of “green” fluorescence.

Such sigmoidal dependence between the signal intensity and analyte concentration can be described statistically by utilizing the four-parameter logistic (4PL) curve for calculating the analytical limit of detection (LOD) of the method [79,80]. In the absence of an exact binding model, we used a similar approach with the 4PL, Equation (1), to obtain characteristic parameters of our detection method:

(1) $$I=I_{B}+\frac{I_{MAX}-I_{B}\;}{1+\left(10^{\log\left(EC50\%\right)-Conc}\right)*HC}$$

Here, I_MAX_ and I_B_ are signal intensity value at the top of the curve and at the bottom respectively, log(EC50%) is the signal intensity value that produces 50% signal response, and HC is the slope-like parameter often referred to as the Hill coefficient [79,80]. The limit of detection (LOD) was then calculated based on the value of three times the standard deviation of the blank value, 3σ_blank_, obtained from the fit. Based on such estimate, the LOD of the method results in 1.6 × 10^−6^ M. Similar results can also be obtained using simple 3:1 signal to noise ratio considerations amounting to micromolar detectible concentration of the analyte. If we further consider that our sample volume is 60 microliters, the estimate suggests the detection limit of the method to be just below one picomole of the target miR-21 molecule. The limit of detection could be lowered using previously suggested sensitive laser spectroscopic methods [81]. Clearly, individual AgNCs can be observed using Total Internal Reflection Fluorescence (TIRF) microscopy, providing the excitation line is carefully chosen to match the appropriate excitation band of AgNCs. Other optimization protocols for the volumetric detection as in our study include running the assay at a lower concentration with voltage boost on the photomultiplying tube (PMT) of the detector, or other dedicated specific hardware enhancements may be considered. Indeed, we have previously shown that “green” fluorescence peaks can be analyzed in detail with PMT voltage enhancements [71]. Figure S1 shows a detailed analysis of the “green” emission region, revealing the cluster of green fluorescence peaks with at least two excitation-emission maxima: λ_EXC_/λ_EM_ = 460/565 nm and λ_EXC_/λ_EM_ = 480/565 nm. The fine structure of the “green” cluster of peaks indicates two major routes for the excitation of the “green” emission at λ_EM_ = 565 nm. We chose to follow the emission peak at λ_EXC_/λ_EM_ = 480/565 nm, as it displays a larger change in intensity with the concentration of added miR-21 than the emission peak at λ_EXC_/λ_EM_ = 460/565 nm. Figure S1 shows the comparative analysis of both peaks as well as their optical response to the added target miR-21. Although the λ_EXC_/λ_EM_ = 480/565 nm outperforms λ_EXC_/λ_EM_ = 460/565 nm peak for the design we used in this study, we can speculate that for some miR target sequences and other loop designs, the λ_EXC_/λ_EM_ = 460/565 nm peak may be the dominant de-excitation route [71]. In that case, this latter peak must be chosen as the default detection read-out signal.

The changes in fluorescence of AgNCs when the C_12_-loop-miR21-probe binds the miR-21 target sequence are very noticeable. These changes are not restricted to the visible range of emission wavelengths only. Figure S2 shows changes taking place upon addition of miR-21 in the UV/UV range (220–300 nm/270–400 nm). The UV emission that is excited in UV shows a gradual increase in intensity with well-separated peaks. Therefore, another alternative to using the “green” emission is to monitor changes of fluorescence that appear in the UV region. Alternative detection strategies are described in detail in the Supplementary Materials. Our results show that the UV emissive states also can be used for the detection of miR sequences. However, the detailed quantitative analysis of miR quantities present in a sample are better analyzed using “green” emissive states, as they greatly outperform “UV” emissive states in relative changes of emission intensity.

We tested the specificity of the C_12_-loop-miR21-probe towards the miR-21 target sequence by using other RNA sequences. First, miR-21 was scrambled to remove complementarity towards the stem’s binding domain. The scrambled miR21 sequence has the same number of specific bases as miR-21, including three rC’s, five rG’s, eight rU’s, and six rA’s. Figure S3 shows the 2D spectra of fluorescence in the visible region upon progressive addition of molar equivalent of scrambled miR-21 to C_12_-loop-miR21-probe. Only minor changes were observed in both “red” and “green” fluorescence peaks. Additionally, we tested the response of AgNC/ C_12_-loop-miR21-probe to the presence of miR-25 molecule. Figure S4 shows the results obtained with miR-25. Again, only minor changes of fluorescence intensity were observed upon addition of up to 1.25 molar equivalent of miR-25. These results suggest that the changes in the green emission we observe with miR-21 are indeed due to specific complementary binding of miR-21 to AgNC/ C_12_-loop-miR21-probe.

### 2.5. Possible Mechanisms Involved in miR21 Detection Using Fluorescence of AgNCs Templated by C_12_-Loop-miR21-Probe

To make an efficient detection system, changes of observable properties need to be apparent. Fluorescent AgNCs have gained popularity as optical entities for sensing applications due to several advantages over regular molecular fluorophores including high sensitivity of fluorescence to subtle changes in the environment. Our sensing design involves looped structures of a hairpin, where the stem of the hairpin contains a complementary sequence to a miR sequence of interest. The idea is that the loop is a selective host of AgNCs and the disruption of the hairpin structure results in dramatic changes of AgNCs surroundings and as a result AgNCs fluorescent properties. The observable optical properties change in response to the amount of miR-21 present in the sample. It seems that the response of the fluorescence intensity is strongly dependent on the molar equivalency of the miR-21 relative to the C_12_-looped hairpin (M_miR21_/M_AgNC_).

Indeed, C_12_ looped hairpin structures host nanoclusters with the “red” emission, which is similar to the emissive pattern we observed for other C_12_ templates including C_12_-alone, ssDNA-C_12_, and dsDNA-C_12_ templates [2]. For the detection of miR sequences involving fluorescent silver nanoclusters, the sensing ability is tightly linked to the appearance of “green” emitting species, as we observed in this study. The intensity of the “green” fluorescence increases dramatically to very bright green emissions that dominate the fluorescence spectrum. What is the possible mechanism of sensing by AgNC? Why does the green fluorescence appear? The following are possible mechanisms of such a phenomenon.

Silver nanoclusters are generally considered to have a rod-like shape with DNA favoring such selective formation of the elongated shapes. The rod-like cluster core has been suggested to be one of the common features that fluorescent DNA templated AgNC share, despite wide differences in emissive properties. The detailed crystal structure of the 16 atom AgNC obtained recently confirmed that this generally presumed shape is the true shape of the nanoclusters [82]. Slight changes in the shape of the clusters from rod like to curved (angled, tilted) ones may result in changes of the fluorescent properties [83,84,85]. Such structural changes may occur for the clusters of the same length (Ag_N_) greatly affecting spectral changes of the AgNCs. It was shown that fluorescent properties can be very sensitive to the geometry of the elongated clusters [85]. The fluorescent properties are especially sensitive to how the core of neutral silver atoms (Ag^0^) is arranged within the cluster [83]. It is quite possible that structural changes in the template structure upon binding of the complementary RNA strand may result in changes of AgNC shape from rod-like to curved-like and, thus, their emissive properties from “red” to “green”.

Alternatively, we must consider that the loop opens up during the detection process, exposing AgNCs nested in the loop to aqueous environment. Dielectric properties (ε) of the surroundings play an important role in the spectral characteristics of fluorescent materials including silver nanoclusters [83]. Such change in the environment from mostly non-polar (DNA bases) to mostly polar (H_2_O) may significantly alter effective dielectric constant and thus energetic position of HOMO and LUMO resulting in solvatochromic shift of the emission wavelength from “red” to “green”. Such an environmental change may cause the wavelength shift. The shift turns out to be beneficial for the detection strategies which involve a hairpin loop opening when miR sequences bind, as we demonstrate in this study on the miR21 example.

It is generally accepted that the neutral core dictates the emissive properties of a nanocluster. The magic numbers, 6 and 4, were assigned to “red” and “green” emissive species respectively. A cluster of the following composition $\left[Ag_{6}^{0}Ag_{N-6}^{+}\right]$ containing six neutral silver atoms would emit in “red” and the cluster $\left[Ag_{4}^{0}Ag_{N-4}^{+}\right]$ with four neutral silver atoms would emit in “green” [86]. Conversion of $\left[Ag_{6}^{0}Ag_{N-6}^{+}\right]$ cluster to $\left[Ag_{4}^{0}Ag_{N-4}^{+}\right]$ would result in respective change of color from mostly “red” to “green”. As the clusters are encapsulated in a C_12_ loop, there is a negligible chance for the exchange of the materials, such as the binding of an extra silver atom or the dissociation of one. Therefore, we propose that the changes in the AgNC structure/composition simply occur by changing the oxidative state of the core from $\left[Ag_{6}^{0}\right]\;to\;\left[Ag_{4}^{0}\right]$. It has been recently proposed that such a conversion is possible [87]. The opening of the loop and the exposure of the clusters to larger fresh volumes of solvent would result in more contact with oxidizing species dissolved in the solution such as, for example, molecular oxygen [88]. Therefore, the opening of the loop should trigger conversion of $\left[Ag_{6}^{0}\right]$ to $\left[Ag_{4}^{0}\right]$, and, thus, a change of color form “red” to “green” for the clusters.

While all the above noted mechanistic descriptions may indeed be possible, the most probable explanation that we propose is the activation of dark “green” fluorescent states upon opening of the loop during the binding of miR. The appearance of the “green” fluorescent color is most likely the result of structural changes that activate emission transitions between the states previously appearing “dark” in the spectrum. These transitions may simply be favorable upon the newly exposed polar (water) environment stabilizing the fluorescence of previously “dark” transitions. Such states have been observed before in silver nanoclusters formed using longer Calf-Thymus DNA template [89]. The formation of the non-emitting long-lived transient species was estimated at high efficiency ~25%. The origin of the AgNC-DNA complex was presumably assigned to the charge–transfer nature, explaining the high yield of the non-emitting states.

## 3. Materials and Methods

### 3.1. Materials

All DNA oligonucleotides and RNA sequences were purchased from Integrated DNA Technologies, Inc. (Coralville, IA, USA) as desalted products and used without further purification (Table 1). Sodium borohydride was purchased from TCI America, Inc. (Portland, OR, USA), all other reagents were purchased from Sigma-Aldrich, Inc. (St. Louis, MO, USA). 

### 3.2. Synthesis of Ag-DNA Nanoclusters

In a typical preparation, DNA and AgNO_3_ aqueous solutions were mixed at 55 °C and incubated for 25 min at room temperature in the ammonium acetate buffer (15 mM ammonium acetate, NH_4_OAc, and 5 mM sodium chloride, NaCl). Next, NaBH_4_ aqueous solution was added and stirred vigorously. The final concentrations of the components were *C*_DNA-template_ = 10 μM, *C*_AgNO3_ = 100 nM, *C*_NaBH4_ = 10 μM, *C*_NH4Ac_= 50 mM. The solution then was allowed to react in the dark for 24 h at 4 °C.

### 3.3. Fluorescence Measurements

The excitation and emission spectra were acquired on a Cary Eclipse Fluorescence Spectrophotometer (Agilent Technologies, Santa Clara, CA, USA). In all the measurements concentration of DNA was kept the same at 10 µM. Relative amounts of miR-21 (0, 0.25, 0.5, 0.75, 1.0, and 1.25 molar equivalent of miR-21 to C_12_-loop-miR21-probe) were added to a 60 µL volume of matured AgNC probe and fluorescence was measured after 2 h of incubation. Measurements have been carried out at room temperature of ~22 °C using Sub-Micro Fluorometer Cell, model 16.40F-Q-10 (from StarnaCells, Inc., Atascadero, CA, USA). The excitation–emission matrix spectra (EEMS) were recorded with 2 nm resolution. Fluorescence spectra were recorded with the emission wavelength ranging from 270 nm to 800 nm, and the initial excitation wavelength was set to 220 nm and the final excitation wavelength was set to 700 nm with an increment of 2 nm. The slits were open to 10 µm and the PMT voltage was set to 700 V. Matrix data were then used for 2D contour plot using MagicPlot Pro software.

## 4. Conclusions

In this work, we developed a novel method to detect miR sequences with the use of silver nanoclusters. The proof of principle was demonstrated on detection of miR-21 molecules but can be easily generalized to other miR sequences. The design of the miR probe involves a hairpin DNA structure with a C_12_ loop that templates a specific type of AgNCs. These AgNCs emit in red when the hairpin is closed but activate “green” and “ultraviolet” emitting states of the AgNCs when the hairpin opens due to binding of the target miR molecule. Both “green” and “ultraviolet” states are dark and initially fluorescently silent. Their activation due to binding is so strong that, as our results demonstrate, they can be used for the quantitative detection of miR-21. An important feature of the developed probe is its performance at room temperature, which does not require any additional hardware to control temperature. The experimental results presented here point to a complexity of AgNCs optical properties and future studies will require to pinpoint the exact nature of the differently “colored” emissive species and their subtle responses to environmental changes. Nevertheless, this study demonstrates that using fluorescent AgNCs represents a simple and cost-effective method of miR detection. Considering that fluorescent DNA-AgNCs have emerged as better alternatives to standard organic or QD emitters, this method has a potential for the quantitative analysis of low levels of miR sequences, which will contribute to the establishment of early diagnostic tools in various types of cancer.

## Figures and Tables

**Table 1:** Nucleic acid sequences used for the probe design and as target analyte molecules.

Name	Sequence
C_12_-loop-miR21-probe	5′-TCAACATCAGTCTGATAAGCTACCCCCCCCCCCCTAGCTTA-3′
miR-21	5′-rUrArGrCrUrUrArUrCrArGrArCrUrGrArUrGrUrUrGrA-3′
miR-21 scrambled	5′-rArCrUrGrUrCrArUrUrCrArGrUrArGrUrGrArArGrUrU-3′
miR-25	5′-rCrArUrUrGrCrArCrUrUrGrUrCrUrCrGrGrUrCrUrGrA-3′

## Supplementary Materials

Supplementary materials are available online.
