# Supplementary material for: Micro RNA Sensing with Green Emitting Silver Nanoclusters
Source: Molecules. 2020 Jul 2;25(13):3026. doi: 10.3390/molecules25133026 (PMC7411700; doi:10.3390/molecules25133026)
Supplement: Supplementary file 1 [file molecules-25-03026-s001.pdf]

Supporting Information

Micro RNA sensing with green emitting silver nanoclusters.

Liam E. Yourston[1] and Alexey V. Krasnoslobodtsev[1,*]

1 Department of Physics, University of Nebraska Omaha, Omaha, NE 68182, USA

* Send the correspondence to:

Alexey V. Krasnoslobodtsev, Ph.D.

Department of Physics
University of Nebraska at Omaha
6001 Dodge Street
Omaha, NE 68182, U.S.A.
402-554-3723 (office)
Email: akrasnos@unomaha.edu

S.1: Alternative detection strategy of miR sequences using C_{12}-loop-miR21-probe.

Using alternative "green" peaks.

The intensity of the "green" fluorescence and its dominating feature become apparent at higher concentration of added miR-21. Figure S1 shows the "green" region of AgNCs/C_{12}-loop-miR21-probe at 1.25 molar equivavalent of miR-21. Two major peaks are obvious in the emission with $\lambda_{EXC}/\lambda_{EM}$ as following: i) 460 nm/565 nm and ii) 480 nm/565 nm. Therefore, it appears that addition of miR-21 to AgNCs/C_{12}-loop-miR21-probe produces AgNCs with two emissive states in the "green" region. The 480 nm/565 nm peak is more prominent than the 460 nm/565 nm peak. We conclude, thus, that peak 2 ($\lambda_{EXC}/\lambda_{EM}$ = 480/565 nm) is a preferable way of de-excitation for the AgNC/Ring-d$C_{12\text{-OUT}}$ showing larger emission intensity than peak 1 ($\lambda_{EXC}/\lambda_{EM}$ = 460/565 nm). Although that is the case, both peaks can be potentially used for detection of miR sequences as both show the intensity growth that is miR-21 concentration dependent.

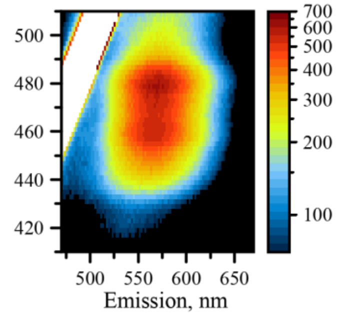

Figure S1. Detailed excitation-emission map of the "green" emission region for AgNC/C_{12}-loop-miR21-probe. Range of excitation wavelengths: 410-510 nm; range of emission wavelengths: 480-680 nm.

Using alternative "ultraviolet" peaks.

Using UV emissive states of the C12-loop-miR21-probe: Figure S2 shows UV/UV Excitation/Emission Maps. While visible peaks both red and green show a complex pattern and merge together in a complicated fashion. Therefore, it makes it challenging to use visible emission with UV excitation for quantitative analysis of miR. On the other hand, UV excited/UV emission shows gradual increase in intensity with well separated peaks. We have explored the possibility of using UV fluorescence also as alternative to "green" emission for detection of miR molecules. UV emission peaks dramatically change (Figure S2, A-B) from 0 molar equivalent to 1 molar equivalent of added miR-21. While the effect we observe is not very strong we hypothesize that assembling AgNC in patterned arrays may provide certain advantage from the order of the structure, boosting thus the detection capabilities towards specific NA sequences. There have been several strategies put forward for detection of nucleic acid sequences. All of the strategies rely on binding to complementary sequence and either enhancing or quenching fluorescence. Although, one might think that a detection strategy for miR or a DNA sequence is easy to devise owing to the high sensitivity of AgNC's to environmental changes, we demonstrate here that this task requires meticulously planned and executed design. The results indicate that the optical response of "green" emitting AgNCs using C_{12}-loop-miR-probe outperforms both "red" AgNCs/C_{12}-loop-miR-probe and "ultraviolet" AgNCs/C_{12}-loop-miR-probe emissive states.

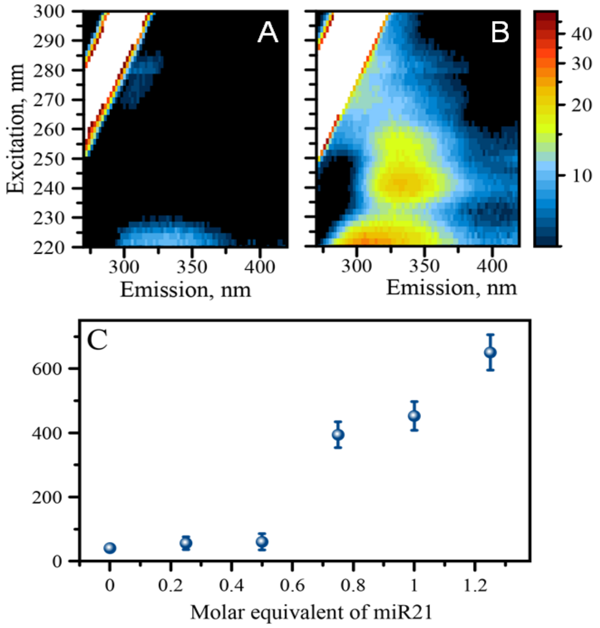

Figure S2. Detailed excitation-emission map of the UV spectral range, 270-420 nm, for AgNC/C_{12}-loop-miR21-probe excited at 220-300 nm range of wavelengths with A) 0 molar equivalent of added miR-21 and B) 1 molar equivalent of added miR-21. C) Fluorescence intensity plot, $\lambda_{EXC}/\lambda_{EM}$ = 240/335 nm, as a function of miR21 molar equivalent concentration, error bar represents variations based on three independent measurements.

S.2: Testing specificity of C12-loop-miR21-probe.

Testing specificity with miR-21 scrambled sequence.

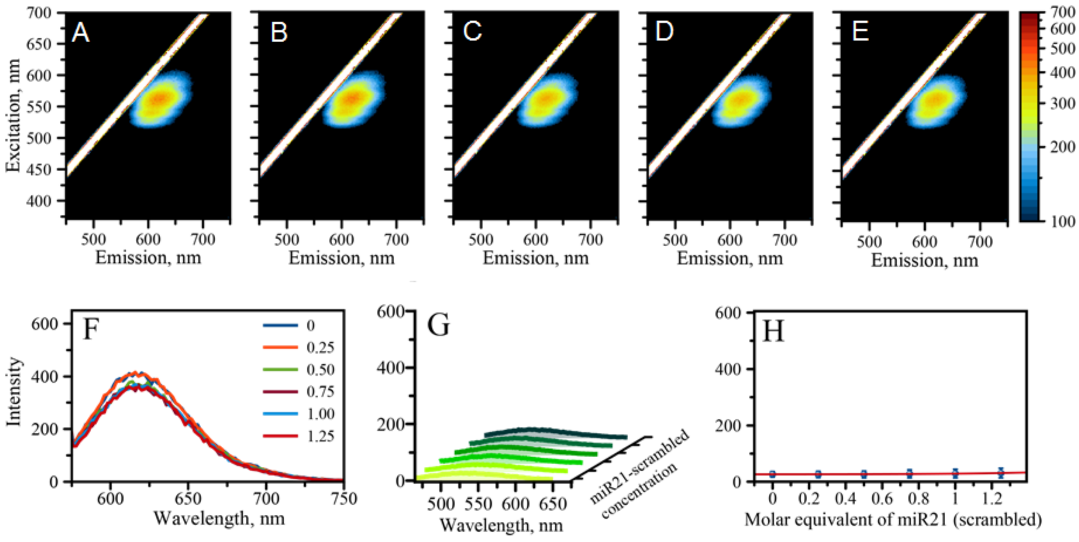

Figure S3. The collection of 2D spectra showing changes upon progressive addition of molar equivalent of scrambled miR-21 sequence to C_{12}-loop-miR21-probe with AgNCs. A) 0 molar equivalent; B) 0.25 molar equivalent; C) 0.50 molar equivalent; D) 0.75 molar equivalent; and E) 1.00 molar equivalent. F) Comparative graph of emission spectra for the "red" AgNCs excited at λ=560 nm showing spectral changes upon incremental addition of molar equivalent of scrambled miR-21 to AgNC/C_{12}-loop-miR21-probe; G) Comparative graph of emission spectra for the "green" AgNCs excited at λ=480 nm showing spectral changes upon incremental addition of molar equivalent of scrambled miR-21 to AgNC/C_{12}-loop-miR21-probe; C) Fluorescence intensity plot, $\lambda_{EXC}/\lambda_{EM}$ = 480/565 nm, as a function of scrambled miR21 molar equivalent concentration showing almost no change in intensity, error bar represents variations based on three independent measurements.

Testing specificity with miR-25 molecule.

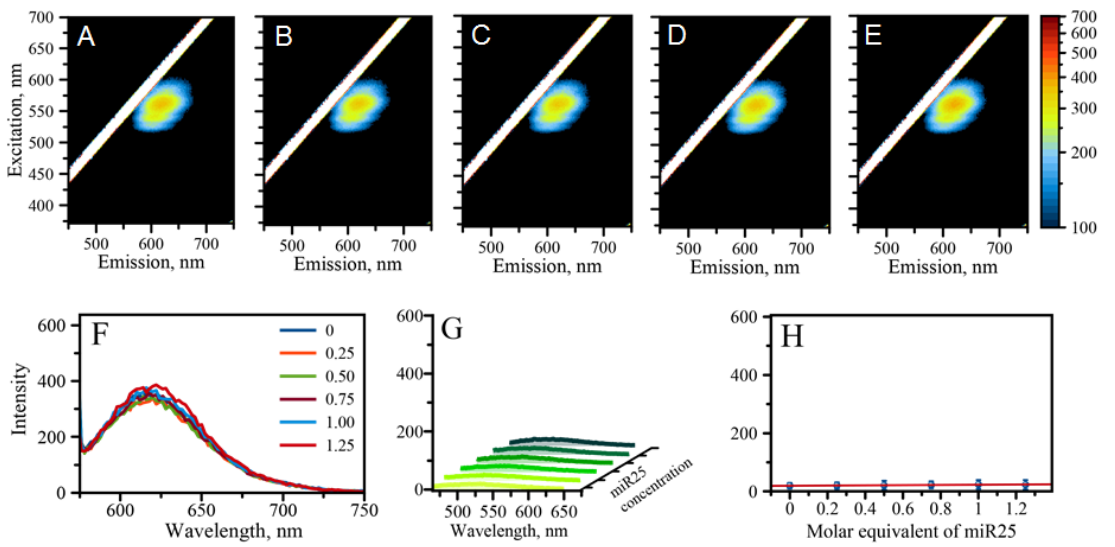

Figure S4. The collection of 2D spectra showing changes upon progressive addition of molar equivalent of scrambled miR-21 sequence to C_{12}-loop-miR21-probe with AgNCs. A) 0 molar equivalent; B) 0.25 molar equivalent; C) 0.50 molar equivalent; D) 0.75 molar equivalent; and E) 1.00 molar equivalent. F) Comparative graph of emission spectra for the "red" AgNCs excited at λ=560 nm showing spectral changes upon incremental addition of molar equivalent of miR-25 to AgNC/C_{12}-loop-miR21-probe; G) Comparative graph of emission spectra for the "green" AgNCs excited at λ=480 nm showing spectral changes upon incremental addition of molar equivalent of miR-25 to AgNC/C_{12}-loop-miR21-probe; C) Fluorescence intensity plot, $\lambda_{EXC}/\lambda_{EM}$ = 480/565 nm, as a function of miR25 molar equivalent concentration showing almost no change in intensity, error bar represents variations based on three independent measurements.
